# Contribution of cardiovascular comorbidities to Alzheimer’s Disease (AD) among older adults with low plasma AD biomarker level

**DOI:** 10.1002/alz.092975

**Published:** 2025-01-09

**Authors:** Yian Gu, Aanya Bahl, Lawrence S. Honig, Min Suk Kang, Richard Mayeux

**Affiliations:** ^1^ Taub Institute for Research on Alzheimer’s Disease and the Aging Brain, Columbia University, New York, NY USA; ^2^ Department of Epidemiology, Mailman School of Public Health, New York, NY USA; ^3^ Taub Institute for Research on Alzheimer’s Disease and the Aging Brain, Vagelos College of Physicians and Surgeons, Columbia University, New York, NY USA; ^4^ Department of Neurology, Vagelos College of Physicians & Surgeons, Columbia University, New York, NY USA

## Abstract

**Background:**

There has been a great advancement in clinical application of blood‐based biomarkers of Alzheimer’s Disease (AD) over the past years. We aimed to examine whether cardiovascular comorbidities may contribute to clinical cognitive impairment in individuals with low level of these biomarkers.

**Method:**

The current cross‐sectional study included 969 elderly [562 cognitively unimpaired, 407 cognitive impairment (104 amnestic MCI and 303 AD)] participants of the Washington Heights, Hamilton Heights, and Inwood Columbia Aging Project (WHICAP) study. We used Quanterix SIMOA HD‐X platforms to measure phosphorylated Tau‐181 (P‐tau181), total (T‐tau), Amyloid beta (Aβ) 40, Aβ42, Glial Fibrillary Acid Protein (GFAP), and Neurofilament Light Chain. Individuals were characterized as ‘biomarker positive’ or ‘biomarker negative’ based on combined GFAP and P‐tau181/Aβ42 cut scores. Clustering of cardiovascular comorbidities was defined as the total number of conditions including diabetes, hypertension, heart disease, and stroke. The association between the cardiovascular comorbidities and cognitive impairment was examined using logistic regression, adjusted for age, sex, ethnic group, and years of education.

**Result:**

Among individuals with 0 ‐ 4 cardiovascular comorbidities, 20%, 43%, 42%, 52%, and 70% had cognitive impairment, respectively (p<0.001). Individuals with more cardiovascular conditions were more likely to be Hispanics, but not different in biomarker positivity status, sex, age, or education. Based on GFAP and P‐tau181/Aβ42, 188 (19.4%) were biomarker positive. As expected, being biomarker positive was associated with higher odds (OR=2.0, 95%CI=1.3‐3.2, P=0.002) of having cognitive impairment, after adjusting for age, sex, ethnic group, and years of education, and comorbidities score. Still, 37% of the 781 biomarker negative individuals had cognitive impairment. We further found that among these biomarker negative individuals, having more comorbidities (OR=1.40, 95%CI=1.02‐1.92, P=0.035) was associated with higher odds, while education was associated with lower odds (OR=0.80, 95%CI=0.75‐0.85, P<0.001), of having cognitive impairment. No association of comorbidity and cognitive impairment (OR=1.2, 95%CI=0.68‐2.15, P=0.52) was found in biomarker positive individuals.

**Conclusion:**

Plasma AD‐related biomarkers were associated with clinical cognitive impairment. Older adults with low level of AD biomarkers may still be at risk of developing cognitive impairment if they have high burden of cardiovascular comorbidities.

